# Ameliorative effects of histidine on oxidative stress, tumor necrosis factor alpha (TNF-α), and renal histological alterations in streptozotocin/nicotinamide-induced type 2 diabetic rats

**DOI:** 10.22038/ijbms.2020.38553.9148

**Published:** 2020-06

**Authors:** Maryam Nasri, Sina Mahdavifard, Esmaeel Babaeenezhad, Glavizh Adibhesami, Negar Nouryazdan, Saeid Veiskarami, Sobhan Rahimi Monfared, Mehdi Birjandi, Hassan Ahmadvand

**Affiliations:** 1 Department of Biochemistry and Genetics, Lorestan University of Medical Sciences, Khorramabad, Iran; 2 Growth and Development Research Center, Tehran University of Medical Science, Tehran, Iran; 3 Department of Clinical Biochemistry, Faculty of Medicine, Ardabil University of Medical Sciences, Ardabil, Iran; 4 Department of Clinical Biochemistry, School of Medicine, Student Research Committee, Shahid Beheshti University of Medical Sciences, Tehran, Iran; 5 Department of Clinical Biochemistry, Faculty of Medicine, Lorestan University of Medical Sciences, Khorramabad, Iran; 6 Agricultural and Natural Resources Research and Education Center, Department of Animal Science, Lorestan, Iran; 7 Nutritional Health Research Center, Lorestan University of Medical Sciences, Khorramabad, Iran; 8 Razi Herbal Medicines Research Center, Faculty of Medicine, Lorestan University of Medical Sciences, Khorramabad, Iran

**Keywords:** Diabetic nephropathy, Gene expression, Histidine, Inflammation, Oxidative stress, Type 2 diabetes mellitus

## Abstract

**Objective(s):**

The present study sought to evaluate the beneficial effects of histidine (His) on oxidative stress, tumor necrosis factor alpha (TNF-α), renal histological alterations and anti-oxidant enzymes gene expressions in type 2 diabetic rats.

**Materials and Methods:**

Streptozotocin/nicotinamide (STZ/NA) induced diabetic rats were used as an animal model of type 2 diabetes. One group of rats received daily His (1000 mg/l) in drinking water for 8 weeks, whereas other groups (control and untreated diabetic groups) received only water. Different parameters such as glucose, insulin, insulin resistance, lipid profile, cardiac risk ratios, renal functional markers, and oxidative stress were determined in all groups. Moreover, renal histological alterations, mRNA expressions of anti-oxidant enzymes, and TNF-α were evaluated in the rats.

**Results:**

His exhibited a protective effect on glucose, insulin, insulin resistance, lipid profile, cardiac risk ratios, renal functional markers, oxidative stress, and TNF-α. Furthermore, His restored the renal histological alterations and normalized the augmented mRNA expressions of renal anti-oxidant enzymes (glutathione peroxidase (GPX) and Cu-Zn superoxide dismutase (Cu-Zn SOD)) and TNF-α.

**Conclusion:**

His could ameliorate diabetes complications related to oxidative stress, inflammation, dyslipidemia, hyperglycemia, insulin resistance, and nephropathy. Hence, the use of this amino acid is recommended for diabetic patients in order to reduce diabetes complications.

## Introduction

As a metabolic disorder, Type 2 diabetes mellitus (T2D) is one of the most significant global health problems. About 382 million people suffered from T2D in 2013. The number of people affected by T2D is estimated to reach 592 million in 2035. The most common type of diabetes mellitus (DM) is T2D, which is illustrated by diminished secretion of insulin, insulin resistance, and β-cell dysfunction. Thirst, polyuria, and fatigue are the most common clinical signs of this disease (1-3).

Various organs and tissues are impaired by hyperglycemia. Hyperglycemia induces mitochondrial dysfunction through the increment of nicotinamide adenine dinucleotide (NADH) and Flavin adenine dinucleotide (FADH2) levels and prevents proton receiving by complex III. These events subsequently lead to reactive oxygen species (ROS) generation and increase oxidative stress (4, 5). ROS can ultimately cause cellular apoptosis and tissue destructions through their ability to damage biomolecules, including DNA, lipids, and proteins (6). On the other hand, high levels of glucose also trigger cellular damages through direct reactions with macromolecules. The increment of lipid peroxidation and the glycation of anti-oxidant enzymes disrupt the anti-oxidant system (7-10). Furthermore, it has been proposed that hyperglycemia contributes to the development of inflammation. Inflammatory cytokines such as TNF-α play a major role in insulin resistance. Likewise, they are involved in diabetic complications such as diabetic nephropathy (11-13). Nevertheless, the application of compounds with anti-oxidant and anti-inflammatory characteristics seems to be an efficient strategy to counteract diabetic injuries. In recent years, researches have focused on the significance of amino acids in preventing DM complications (14, 15).

Histidine (His) is a semi-essential amino acid in the body capable of having different roles such as acting as a buffer, binding to divalent metal ions, reducing insulin resistance, and scavenging of free radicals. His also has anti-oxidant, anti-inflammatory, anti-glycation, and anti-lipid peroxidation properties (16-19). It has been demonstrated that the low level of His is related to increased inflammation and oxidative stress in chronic kidney diseases (20). A review of the previous studies indicates that some researchers have found decreased plasma His in diabetic mice compared with the controls (19). 

Due to the important role of increased oxidative stress and inflammation in the pathophysiology of DM and the valuable properties of His in the inhibition of oxidative damages, glycation, and reduction of insulin resistance, this study was carried out to evaluate the effects of His on oxidative stress biomarkers, lipid profile, cardiac risk ratios, insulin, insulin resistance (HOMA-IR), renal histological alterations, and the renal gene expressions of anti-oxidant enzymes and TNF-α in Wistar rats with streptozotocin/nicotinamide (STZ/NA)-induced T2D.

## Materials and Methods

***Chemicals***

STZ, NA, and L-Histidine were purchased from Sigma Aldrich Company (USA). Tris-Ethylenediaminetetraacetic acid (Tris-EDTA), Tris-Hcl and EDTA were obtained from Merck Company (Germany). Commercial kits for the evaluation of fasting blood sugar (FBS), triglyceride (TG), cholesterol (Chol), high density lipoprotein cholesterol (HDL-C), uric acid (UA), and creatinine (Cr) were purchased from Pars Azmoon Company (Tehran, Iran). Also, Asanzol (Trizol) reagent and commercial kits for the biochemical assessment of malondialdehyde (MDA), glutathione (GSH), catalase (CAT), glutathione peroxidase (GPX), total superoxide dismutase (SOD), nitric oxide (NO), and myeloperoxidase (MPO) were purchased from Aryagen Sobhan Azma Novin (Asan) Company (Khorramabad, Iran).

***Animals and experimental design***

Thirty adult male Wistar rats (180–200 g) were procured from Razi Herbal Medicines Research Center, Lorestan, Iran. The rats were housed in a room with standard conditions for laboratory animals including 23±2 ^°^C temperature, humidity of 45% to 55% and 12 hr light/dark cycle. The animals had free access to food pellets except when starvation was required. Moreover, water was provided *ad libitum*. These conditions were provided for the animals at the animal lab of Razi Herbal Medicines Research Center, Lorestan, Iran. All of our experimental procedures and stages of the assessments were performed in conformity with the Research Ethics Committee of the University and Iranian Ethical Guidelines for the Application of Animals in Research. This study also was accepted by the Animal Ethics Committee of Lorestan University of Medical Sciences. Furthermore, our study protocols were in accordance with the methods admitted by the National Institutes of Health (NIH1978). 

The rats were haphazardly divided into three groups (n=10): control group, untreated diabetic (UTD) group, and His-treated diabetic (HTD) group. The rats in the HTD group received daily His (1000 mg/l) (19) in drinking water for 8 weeks after the induction of diabetes.

***Induction of T2D***

For induction of T2D, the animals in the UTD and HTD groups were fasted for 12 hr, while they had free access to water. Nicotinamide (110 mg/Kg, soluble in normal saline) was intraperitoneally injected after the fasting period. The intraperitoneal injection of STZ (65 mg/kg, soluble in citrate buffer (0.05M, pH=4.5)) was accomplished fifteen min after NA injection (21). The induction of hyperglycemia was approved by the measurement of FBS in the blood collected from the tail vein of the rats on the third day after the STZ injection. The animals with an FBS level of more than 250 mg/dl were considered diabetic rats; hence, they were selected for the study (22). The measurement of FBS was performed using a glucometer (Accu Chek Active, Germany).

***Sample collection***

At the end of the eight-week treatment period, the animals in each group were anesthetized by intraperitoneal injection of ketamine and xylazine (13 mg/kg and 87 mg/kg, respectively). Subsequently, blood samples were collected from the animals’ hearts. To clot the blood, the collected blood samples were stored at normal room temperature for 20 min. For the separation of serum, the collected blood samples were instantaneously centrifuged at 3000 rpm for 15 min (4 ^°^C). The serum samples were poured into microtubes and kept at -20 ^°^C until the time of serum biochemical evaluations. Moreover, the livers and kidneys of the animals were removed. The right kidney was divided into 2 equal parts. One part of the right kidney and the liver were immediately homogenized for biochemical measurements. Another part of the right kidney was kept at -70 ^°^C to be used for molecular evaluation and gene expression study. The left kidney was fixed in formaldehyde solution (10%) for histological evaluations. 

***Biochemical study***

*The measurement of FBS, insulin, insulin resistance, lipid profile, and cardiac risk ratios*

The serum levels of FBS, TG, Chol, and high HDL-C were measured by a biochemical auto analyzer (Olympus AU-600, Tokyo, Japan) utilizing commercial kits (Pars Azmoon, Tehran, Iran). 

The levels of low density lipoprotein (LDL) and very low density lipoprotein (VLDL) were determined by the equation proposed by Friedewald *et al.* (23). Moreover, cardiac risk ratio 1 (CRR 1): (TC/HDL-C) and CRR 2: (LDL/HDL-C) were calculated by the equation proposed by Ikewuchi (24). 

The serum level of insulin was measured by enzyme linked immunosorbent assays (ELIZA) technique using the rat insulin kit based on the manufacturer’s protocol (Mercodia AB, Sylveniusgatan 8A SE-754 50 Uppsala, Sweden). HOMA-IR, as the marker of insulin resistance, was calculated using the equation below: 

HOMA-IR=[(fasting insulin (U/ml)×fasting glucose [mM]) /22.5].

*The measurement of renal functional markers*

The levels of renal functional markers, including UA and Cr, in the sera of animals, were determined by a biochemical auto analyzer utilizing commercial kits (Pars Azmoon, Tehran, Iran). 

*The Evaluation of oxidative stress biomarkers*

The concentrations of MDA, GSH, and NO and the activities of CAT, GPX, total SOD, and MPO in the serum, the kidneys, and livers of the animals were determined by colorimetrical commercial biochemical kits (Asan, Khorramabad, Iran).

***Gene expression study***

*Total RNA isolation and complementary DNA (cDNA) synthesis*

In this study, the gene expression of TNF-α and anti-oxidant enzymes, including Cu-Zn SOD and GPX, were evaluated by real-time quantitative PCR in the kidneys of rats. Total RNA isolation was carried out according to the following steps:

The small part of each frozen kidney sample (100-150 mg) was placed in the microtube containing Asanzol (Trizol) reagent (Asan Company, Khorramabad, Iran). Subsequently, the sample was homogenized by an ultrasonic homogenizer (HIELSHER UP 200H, Germany). The homogenized tissue was incubated at 25 ^°^C (5 min). Then, chloroform in the volume of 200 ml was appended to the microtube and subsequently centrifuged for 15 min (12000× g, 4 ^°^C). 250 µl of the upper phase was transmitted to another sterile microtube. In the next step following the appending of isopropyl alcohol (250 µl), the RNA was precipitated by centrifugation for 15 min (12000× g, 4 ^°^C). RNA precipitations were washed with ethanol (75%) by centrifugation for 5 min (7500× g, 4 ^°^C). After washing, the precipitations were dried in the air under sterile conditions. RNA precipitations were resuspended in diethyl pyrocarbonate-H_2_O (DEPC-H_2O)._

The purity and concentration of RNA precipitation were specified at 260 and 280 nm by a NanoDrop spectrophotometer (Biochrom WPA Biowave II, UK). 

The integrity of isolated RNA was assessed by gel electrophoresis. The conversion of isolated RNA to cDNA was executed using Yekta Tajhiz Azma (YTA) cDNA Synthesis Kit (YT4500, Iran) according to the manufacturer’s guidelines.

*Real-time polymerase chain reaction (Real-time PCR)*

Real time quantitative PCR was conducted in order to reveal the gene expression levels of GPX, Cu-Zn SOD, and TNF-α in the kidneys via Yekta Tajhiz Azma (YTA) SYBER Green qPCR Master Mix 2x (YT2551, Iran). The reaction was implemented using Rotor-Gene 6000 (Corbett Research). The final volume of each reaction was 20 µl containing Master Mix (10 µl), cDNA (1 µl), specific forward & reverse primers (0.8 µl; Sinaclon, Iran), and distilled water (8.2 µl). 

The sequence of utilized primers (Table 1) for each gene was determined based on the data available in the public database of the Gene Bank of the National Center for Biotechnology Information (NCBI). All reactions were executed in triplicate. Thermal cycling conditions were provided as follows: 1 cycle of 95 ^°^C for 3 min and 40 cycles of 95 ^°^C for 5 sec (denaturation), and 40 cycles of 60 ^°^C for 20 sec (annealing & extension). β-actin was used as the housekeeping gene in this study in order to normalize the data. Negative controls without cDNA were used in all reactions. The standardization curve was structured by drawing the threshold cycle versus serial dilution made of control cDNAs. Product length and the specificity of reaction were examined by agarose gel electrophoresis (1%). 

***Histological assessment***

The kidney samples were processed after their fixation in formaldehyde solution (10%). The preparation of the paraffin blocks was performed after tissue processing. Prepared paraffin blocks were randomly cut into sections with 5 μm thickness and stained by periodic Acid-Schiff (PAS). Mean glomerular volume was measured through a digital procedure equipped with motic camera, motic microscope, and the motic image plus software program (version 2). The method of Weibel and Gomez was used for calculation of the mean glomerular volume (VG). VG= area 1.5×1.38/1.01. The numbers 1.38 and 1.01 represent the shape coefficient and the coefficient size distribution, respectively (25). 

Glomerulosclerosis intensity was investigated semiquantitatively by a skilled histologist in a blinded manner. The intensity of each section was determined by considering a score (0-4) for each glomerulus based on the tuft indicating sclerosis: 0=normal glomeruli, 1=0-25% injury, 2=25%–50% injury, 3=50%–75% injury, and 4= over 75% injury. In the stained tissue, sections of each group ≥150 glomeruli were evaluated in this assessment (26).

The evaluation of leukocyte infiltration was similar to our previous study (27). 

***Statistical analysis***

The results were illustrated by mean±SEM. The biochemical and histological evaluations were separately analyzed by one-way analysis of variance (ANOVA). *Post hoc* LSD test was used in order to determine the significant differences between the groups. Real-time PCR outputs were analyzed by rest-RG software. The level of statistical significance was set at *P*-value<0.05.

## Results

***Biochemical results***

As represented in Table 2, the levels of FBS and HOMA-IR were significantly (2.74-fold and 2.40-fold, respectively) higher in the UTD group compared with the control group (*P*<0.001), while the level of insulin (1.22-fold) was remarkably lower in the UTD group compared with the control group (*P*<0.001). His therapy could significantly (21.77% and 18.86%, respectively) mitigate the increment of FBS and HOMA-IR in the HTD group compared with the UTD group (*P*=0.03 and *P*=0.03, respectively). Moreover, His therapy could significantly (10.48%) increase the level of insulin in the HTD group compared with the UTD group (*P*=0.04). 

Table 2 also indicates the status of lipid profile and CRR ratios. The levels of Chol, TG, LDL, VLDL, CRR 1 and 2 significantly (1.37-fold, 1.65-fold, 7.24-fold, 1.65-fold, 2.77-fold and 15.30-fold, respectively) enhanced in the UTD group compared with the control group (*P≤*0.001). His treatment could remarkably (18.26%, 36.12%, 40.22%, 36.12%, 41.96% and 57.28%, respectively) reduce the parameters above in the HTD group compared with the UTD group (*P*=0.01, *P*<0.001, *P*=0.002, *P*<0.001, *P*<0.001, *P*<0.001 and *P*<0.001, respectively). A significant decline (2-fold) in the serum level of HDL-C was observed in the UTD group compared with the control group (*P*<0.001). Treatment with His could significantly (40.71%) augment the serum HDL-C level in the HTD group compared with the UTD group (*P*<0.001). 

The serum levels of UA and Cr, as the renal functional markers, have been indicated in Table 2. A significant increment (1.76-fold and 1.25-fold, respectively) in the levels of UA and Cr was detected in the UTD group compared with the control group (*P*=0.007 and *P*=0.01, respectively). Treatment with His significantly (49.79% and 17.33%, respectively) inhibited the increment of serum UA and Cr in the HTD group compared with the UTD group (*P*=0.002 and *P*=0.008, respectively). His could improve the levels of renal functional markers, so that the levels of UA and Cr in the HTD group reached the normal levels observed in controls (*P*>0.05). 

**Figure 1:**
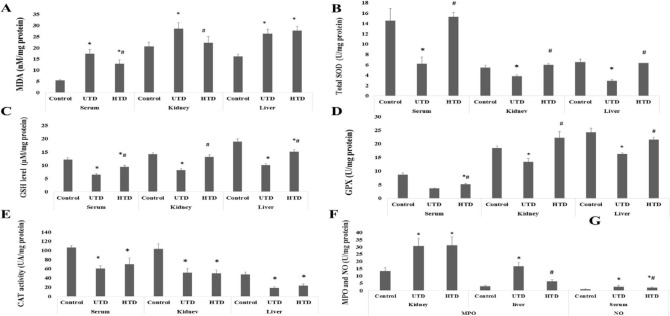
The effects of His therapy on oxidative stress biomarkers including A) MDA, B) Total SOD, C) GSH, D) GPX, E) CAT, F) MPO, and G) NO in different groups. Data are represented as Mean ±SE. * Shows significant difference against the control group; ^#^ Shows significant difference against the UTD group

The effect of His on the levels of MDA in sera, the kidney, and liver have been indicated in Figure 1. MDA levels in serum, the kidney, and liver of the UTD group significantly augmented (3.17-fold, 1.38-fold, and 1.63-fold, respectively) in comparison with the control group (*P*<0.001, *P*=0.02 and *P*<0.001, respectively). In the HTD group, His could significantly (25.53% and 22.03%, respectively) reduce the level of MDA in serum and the kidney compared against the UTD group (*P*=0.02 and *P*=0.04, respectively). There was no significant difference in the level of liver MDA between HTD and UTD groups (*P*>0.05). 

The levels of GSH in serum, the kidney, and liver in the three groups are indicated in Figure 1. GSH levels in serum, the kidney, and liver were significantly reduced (1.86-fold, 1.73-fold, and 1.87-fold, respectively) in the UTD group in comparison with the control group (*P*<0.001). In the HTD group, His could significantly (45.07%, 60.88%, and 50.19%, respectively) enhance GSH levels in serum, the kidney, and liver compared with the UTD group (*P*=0.01, *P*<0.001, and *P*<0.001, respectively). There was no significant difference in the level of the kidney GSH between the HTD and UTD groups (*P*>0.05). 

The activities of CAT in serum, the kidney, and liver have been indicated in Figure 1. CAT activities in serum, the kidney, and liver were significantly (1.74-fold, 2.01-fold, and 2.56-fold, respectively) lower in the UTD group compared with the control group (*P*<0.001). It was observed that there were no significant differences in the activities of CAT in serum, the kidney, and liver between the His treated rats and the untreated rats with T2D (*P*=0.38, *P*=0.93, and *P*=0.39, respectively). 

Total SOD activities in serum, the kidney, and liver have been shown in Figure 1. Total SOD activities in serum, the kidney, and liver of the UTDs were significantly reduced (2.46-fold, 1.44-fold, and 2.23-fold, respectively) compared with the controls (*P*=0.001, *P*<0.001, and *P*<0.001, respectively). In the HTDs, His could notably (144.94%, 57.51%, and 118.90%, respectively) increase total SOD activities of serum, the kidney, and liver compared with the UTDs (*P*=0.001, *P*<0.001, and *P*<0.001, respectively). 

GPX activities in serum, the kidney, and liver are indicated in Figure 1. Serum, renal, and liver activities of GPX in the UTD group were significantly less (2.37-fold, 1.37-fold, and 1.48-fold, respectively) than those of the control group (*P*<0.001). The activities of GPX in the HTD group were significantly higher (40.65%, 60.88%, and 32.22%, respectively) than those of the UTD group (*P*=0.002, *P*<0.001, and *P*<0.001, respectively). 

MPO activity in the kidney and liver of the UTD group (Figure 1) significantly enhanced (3.11-fold and 5.22-fold, respectively) in comparison with the control group (*P*=0.002 and *P*<0.001, respectively). In the HTD group, MPO activity was significantly (63.26%) decreased in the kidney compared with the UTD group (*P*=0.002). There was no significant difference in the activity of liver MPO between UTD and HTD groups (*P*=0.38).

The level of serum NO has been indicated in Figure 1. The serum level of NO in the UTD group was significantly higher (3.17-fold) than that the control group (*P*<0.001). His could significantly (25%) reduce NO concentration in the serum of the HTDs compared with the UTDs (*P*=0.01).

***Histological study***

**Figure 2:**
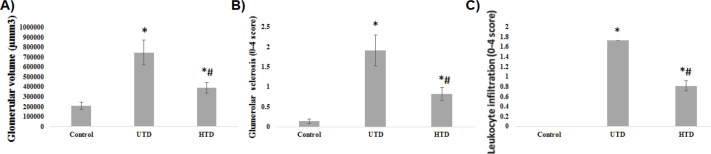
A) Glomerular volume, B) Level of glomerular sclerosis, and C) Level of leukocyte infiltration in different groups. Data are represented as Mean ±SE. *Shows significant difference against the control group. #Shows significant difference against the UTD group

**Figure 3:**
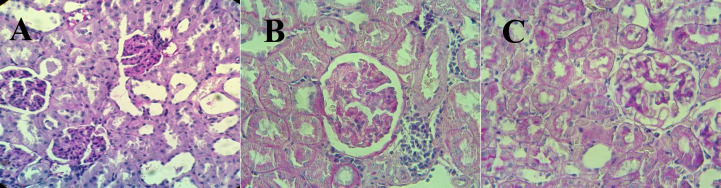
The effects of His therapy on renal histopathological parameters observed in the photomicrographs of kidney sections stained with PAS. A) Micrograph (400×) from the kidney of an animal in the control group demonstrated normal glomeruli and tubules. B) Micrograph (400×) from the kidney of a diabetic untreated animal demonstrated glomerulosclerosis (Score 3) and lymphocyte infiltration. C) Micrograph (400×) from the kidney of a diabetic animal treated with His demonstrated glomerulosclerosis (Score 1) with low level of lymphocyte infiltration

The results of the histological evaluation of PAS stained kidney sections have been shown in Figures 2 and 3. Glomerular volume, glomerular sclerosis, and leukocyte infiltration in the UTD group significantly increased compared with the control group (*P*<0.001). Treatment with His could significantly reduce the aforementioned parameters in the HTD group compared with the UTD group (*P*<0.001).

***Gene expression study***

**Figure 4:**
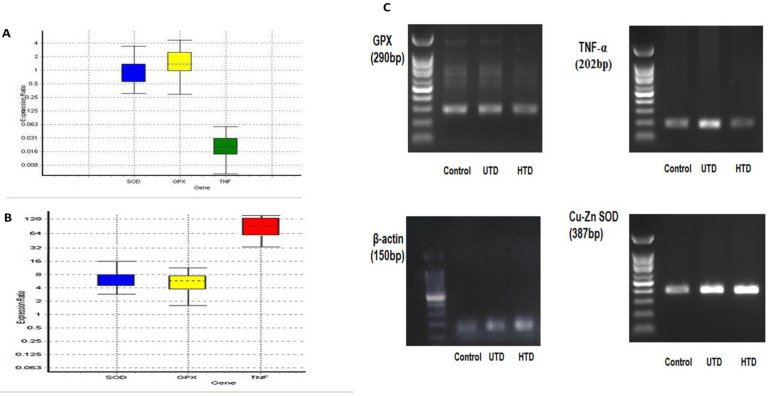
A) Box plot indicates renal gene expression ratio of Cu-Zn SOD, GPX, and TNF-α, in the UTD group. B) Box plot indicates renal gene expression ratio of Cu-Zn SOD, GPX, and TNF-α in the HTD group. Boxes represent the interquartile range or the middle 50% of observations. The dotted line represents the median gene expression. Whiskers represent the minimum and maximum observations. C) Agarose gel electrophoresis (1%) of real time-PCR amplification of β-actin, Cu-Zn SOD, GPX, and TNF-α

The status of Cu-Zn SOD, GPX, and TNF-α gene expressions in the UTD and HTD groups compared with the control group have been indicated in Figure 4, and Tables 3 and 4. A significant increase in the mRNA expression levels of Cu-Zn SOD, GPX, and TNF-α was observed in the UTD group in comparison with the control group (*P*=0.008, *P*=0.012, and *P*=0.002, respectively). In the HTD group, His therapy could restore the mRNA expression of Cu-Zn SOD and GPX to their normal expression levels, so that their expressions did not significantly differ from those in the control group (*P*=0.91 and *P*=0.44, respectively). Furthermore, His therapy significantly reduced TNF-α mRNA expression in the HTD group in comparison with the control group (*P*=0.02).

## Discussion

The ameliorative effects of eight-week His therapy on diabetic complications in STZ/NA**-**induced type 2 diabetic rats have been indicated in the present study. Treatment with His significantly diminished hyperglycemia, insulin resistance, and increment of renal functional markers, inhibited oxidative stress and inflammation, ameliorated lipid profile and insulin secretion, and finally reversed renal histological alterations and gene expressions in STZ/NA**-**induced type 2 diabetic rats. 

The beneficial effects of amino acids in the management of hyperglycemia have been highlighted in certain studies (14, 28). In the present study, we similarly found that His therapy could mitigate FBS in the rats with T2D. Moreover, the hypoglycemic effect of His has been reported in STZ-induced diabetic rats under His or carnosine therapy (19). Additionally, the hypoglycemic property of some amino acids, including glycine (Gly) (14), cysteine (Cys) (28), isoleucine (Ile) (29), and lysine (Lys) (15) have been reported *in vivo*. A published paper indicated that His hypoglycemic action was linked to the impression on the central nervous system (CNS), motivation of signal transducer and activator of transcription 3 (STAT3) pathway, and diminution of the gene expression of gluconeogenic enzymes in the livers of mice (30). Confirming the results of previous studies, the present research exhibits the beneficial effects of amino acids in the management of hyperglycemia. 

The exhibition of the useful effects of His on insulin sensitivity in rats with T2D in this research approves previous studies indicating the increment of insulin sensitivity by amino acids, including His (16, 28). Furthermore, it was reported that insulin resistance is associated with decreased His levels in the plasma, liver, and skeletal muscles in Zucker diabetic fatty rats (31). According to data obtained from the determination of insulin level and HOMA-IR, it could be concluded that in addition to the mechanism affecting CNS which was reported previously, the hypoglycemic effect of His in rats with T2D might be associated with the improved beta cell function. This hypothesis confirms the anti-oxidant and anti-inflammatory characteristics of His and its function regarding the reduced insulin resistance homeostatic model assessment for insulin resistance (HOMA-IR) (16). 

Our results also indicated that the serum levels of Chol, TG, LDL, VLDL, CRR 1, and CRR 2 increased, and HDL-C decreased in type 2 diabetic rats. The changes in these parameters are similar to the previous reports (19, 28, 32). In the HTD group, His could significantly reverse the aforementioned parameters. Moreover, hypolipidemic effects of His have been highlighted in the elderly and the animal model of hepatic steatosis in mice (33-35). Furthermore, the hypolipidemic effects of other amino acids such as glutamine (Gln), aspartic acid (ASP), arginine (Arg), glutamic acid (Glu), Cys, and Lys have been previously reported (15, 28, 36). The mechanism of the hypolipidemic effect of His might be due to its role in the reduction of activity and mRNA expression of malic enzyme, fatty acid synthase, β-hydroxy β-methylglutaryl-CoA (HMG-CoA) reductase, and sterol regulatory element-binding proteins (SREBP) (33). 

Our results revealed that the serum levels of Cr and UA significantly increased in type 2 diabetic group versus the control group similar to previous studies (37). His could significantly diminish the levels of serum Cr and UA in the treated type 2 diabetic rats in comparison with the untreated type 2 diabetic rats. The beneficial effects of some amino acids such as His, L-Gln, and Arg on the renal function have also been reported in published papers (36, 38, 39). The improvement of serum Cr and UA in the HTD group might be due to His effects in the increment of GFR. Unfortunately, collecting enough urine for GFR measurement was one of our limitations.

Hyperglycemia leads to the impairment of various organs and tissues in DM. That is, hyperglycemia not only leads to ROS generation but also suppresses the antioxidant system through the glycation of anti-oxidant enzymes (8). The diminution of anti-oxidant enzyme activities could cause the surplus accessibility of superoxide anion and H_2_O_2_, and also could trigger LPO (40). In the present study, the significant lowering in GSH levels and CAT, total SOD and GPX activities, and the significantly increased MDA levels, as the marker of LPO, in various organs of diabetic rats approve the augmented oxidative stress induced by hyperglycemia. These findings were similar to previous studies (41, 42). Anti-oxidant effects of His have also been indicated in obese women (16), animal model of DM (19), liver damages (43), and Cu-induced oxidative stress (17). Similarly, in our study, His therapy in diabetic animals could enhance the activities of total SOD, GPX, and GSH, and reduce serum and kidney LPO. The recovery of anti-oxidant enzyme activities and GSH levels by His might neutralize free radicals and subsequently suppress reactions leading to LPO (42). 

Diabetic nephropathy (DN) is one of the prevalent diabetic complications in the progressive stage of DM. Renal histopathological alterations typically occur in DN including increment in the thickness of glomerular basement membrane, mesangial expansion, and glomerulosclerosis (44). In the UTD group, a significant increase in mean glomerular volume, glomerular sclerosis, and leukocyte infiltration clearly approved diabetes-induced kidney injuries. 

It has been suggested that endothelial dysfunction (ED) plays a pivotal role in the pathogenesis of DN (45). Myeloperoxidase is proposed as a significant marker for the prediction of ED in patients with T2D (46). Myeloperoxidase decreases the bioavailability of NO and ultimately leads to ED and instability of atherosclerotic plaques (47). Hence, it is a good marker for ED and inflammation. In the present study, increased renal and liver MPO activities in the UTD group indicated enhanced inflammation and risk of ED and renal injuries, which were approved by our histological findings. His therapy significantly reduces renal histopathological damages and renal MPO activity. Confirming the results of previously published papers, this research indicates the renoprotective effects of amino acids such as L-Gln and Arg (38, 39). 

It is widely acknowledged that STZ-induced diabetes triggers an augment in the activity and expression of inducible nitric oxide synthase (iNOS) as the generator of NO (48). In published data, increased iNOS activity and NO level, as the consequences of inflammatory progression, were related to DN (39, 49). Similarly, elevated serum NO was found in our study. The interaction between NO and the superoxide anion leads to nitrosative stress and peroxynitrite generation as an offensive cellular oxidant (50). His therapy could significantly reduce NO levels in diabetic animals similar to previously published data revealing the beneficial effects of amino acids on NO and nitrosative stress (39, 51). 

It has been proven that TNF-α can participate in the pathogenesis of DN through different mechanisms. Some researchers have proposed that pro-inflammatory cytokines, including TNF-α and IL-6, are related to the frequency and intensity of DN (12). In the present study, DM significantly up-regulated TNF-α mRNA expression compared with the control group. In this regard, different studies have demonstrated that DN is associated with the disorders in the balance of cytokines such as amplified TNF-α gene expression (52). His therapy could significantly down-regulate TNF-α mRNA expression in the treated diabetic rats versus the control rats in this study. Similarly, the anti-inflammatory effect of His through down-regulation of TNF-α mRNA expression was reported in mice with colitis (53). The suppressive effects of His on TNF-α expression might be associated with the NF-κB-signaling pathway. Due to its potential function in regulating the transcription process of different cytokines, His might suppress the activation of nuclear factor-κB (NF-κB) and subsequently inhibit the generation of pro-inflammatory cytokines (53). 

Renal GPX gene expression was significantly up-regulated in the UTD group compared with the control group. However, renal GPX activity was significantly reduced in the UTD group. A significant increment in renal GPX mRNA expression that was observed in the UTD group seems to be conquering the decline of GPX activity. Moreover, similar results were reported by other studies (54, 55). His therapy could decrease the renal GPX mRNA expression, and increase its activity in a way that their levels were not significantly different from those in the control group (*P*>0.05). According to these data, the rise of renal GPX activity induced by His might be associated with its effects on the levels of translation and/or post-translational mechanisms. Previous studies ascribed the decline in GPX activity in diabetic rats to post-translational proceedings. Decreased activity might be associated with diabetes-induced oxidative stress and dephosphorylation (54, 56). 

Renal Cu-Zn SOD gene expression significantly up-regulated in the UTD group compared with the control group, while total kidney SOD activity significantly reduced. Similar results were reported by other researchers (58). The possibility of dwindled activity and/or mRNA expression of Mn-SOD enzyme for the decline in total SOD activity should not be discounted. Moreover, the post-translational glycation induced SOD inactivation must be considered (57). Indeed, determination of activity and mRNA expression of renal Mn-SOD could clarify the reason for the declined total kidney SOD activity in type 2 diabetic rats. His therapy could lessen renal Cu-Zn SOD mRNA expression, and at once augment total kidney SOD activity, so that their levels were not significantly different from those in the control group (*P*>0.05). Based on these results, increased total kidney SOD activity might be associated with His effects on the translation and/or post-translational events for Cu-Zn SOD. Moreover, it might be associated with His effects on gene expression and/or translation and/or post-translational events for Mn-SOD. 

**Table 1:** Primer sequences for Cu-Zn SOD, GPX, TNF-α, and β-actin

Gene	**Primers position**	**Primers sequences(5'→3')**	**Product size**
**SOD** **(Cu-Zn)**	Forward	TTCGAGCAGAAGGCAAGCGGTGAA	387
	Reverse	AATCCCAATCACACCACAAGCCAA	
**GPX**	Forward	GGTGTTCCAGTGCGCAGAT	290
	Reverse	AGGGCTTCTATATCGGGTTCGA	
**TNF-α**	Forward	CCGATTTGCCATTTCATACC	202
	Reverse	GAGTCCGGGCAGGTCTACTT	
**β-actin**	Forward	AGCACTGTGTTGGCATAGAGG	150
	Reverse	TATCGGCAATGAGCGGTTCC	

(Cu-Zn) SOD: Cu-Zn Superoxide Dismutase, GPX: Glutathione Peroxidase, TNF-α: Tumor Necrosis Factor Alpha, β-actin: Beta-actin

**Table 2:** The effects of His on the levels of FBS, insulin, HOMA-IR, lipid profile & CRRs (TG, Chol, LDL, VLDL, HDL-C, CRR 1 and 2) and renal functional markers (UA & Cr) in control, UTD, and HTD groups

**Parameter**	**Control**	**UTD**	**HTD**
**FBS (mg/dl)**	104.83 ± 5.99	288.00 ± 29.01*	225.28 ± 4.15*#
**Insulin (μg/L)**	1.75 ± 0.04	1.43 ± 0.04*	1.58 ± 0.03*#
**HOMA-IR**	0.44 ± 0.02	1.06 ± 0.03*	0.86 ± 0.06*#
**Chol (mg/dl)**	65.46 ± 1.55	89.92 ± 4.47*	73.50 ± 3.94#
**TG(mg/dl)**	61.20 ± 2.9	101.45 ± 5.01*	64.80 ± 3.95#
**HDL** **-** **C (mg/dl)**	46.83 ± 0.6	23.36 ± 0.54*	32.87 ± 0.85*#
**LDL (mg/dl)**	6.39 ± 1.76	46.27 ± 4.38*	27.66 ± 3.39*#
**VLDL (mg/dl)**	12.24 ± 0.58	20.29 ± 1.00*	12.96 ± 0.79#
**CRR1 (TC/HDL-C) **	1.39 ± 0.03	3.86 ± 0.20*	2.24 ± 0.14*#
**CRR2 (LDL/HDL-C) **	0.13 ± 0.03	1.99 ± 0.19*	0.85 ± 0.11*#
**UA (mg/dl)**	1.41 ± 0.27	2.49 ± 0.31*	1.25 ± 0.09#
**Cr (mg/dl) **	0.60 ± 0.02	0.75 ± 0.03*	0.62 ± 0.02#

* Shows significant difference against the control group; # Shows significant difference against the UTD group

FBS: Fasting Blood Sugar, HOMA-IR: Homeostatic Model Assessment for Insulin Resistance, Chol: Cholesterol, TG: Triglyceride, HDL-C: High Density Lipoprotein Cholesterol, LDL: Low Density Lipoprotein, VLDL: Very Low Density Lipoprotein, CRR 1: Cardiac Risk Ratio 1, CRR 2: Cardiac Risk Ratio 2, UA: Uric Acid, Cr: Creatinine, UTD: Untreated Diabetic, HTD: His-Treated Diabetic

MDA: Malondialdehyde, GSH: Glutathione, CAT: Catalase, GPX: Glutathione Peroxidase, SOD: Superoxide Dismutase, NO: Nitric Oxide, MPO: Myeloperoxidase, UTD: Untreated Diabetic, HTD: His-Treated Diabetic

UTD: Untreated Diabetic, HTD: His-Treated Diabetic

PAS: Periodic Acid-Schiff, His: Histidine

β-actin: Beta-actin, (Cu-Zn) SOD: Cu-Zn Superoxide Dismutase, GPX: Glutathione Peroxidase, TNF-α: Tumor Necrosis Factor Alpha, UTD: Untreated Diabetic, HTD: His-Treated Diabetic

**Table 3:** Output data from Rest-RG software revealed the level of renal gene expression of Cu-Zn SOD, GPX, and TNF-α between control and UTD groups. The level of significant difference against the control group was set at *P-value<*0.05

**Gene**	**Type**	**Reaction efficiency**	**Expression**	**Std. Error**	**95% C.I.**	**P(H1)**	**Result**
**β-actin**	REF	0.7663	1.000				
**Cu-Zn SOD**	TRG	0.7813	6.392	3.808 - 13.108	3.160 - 15.333	0.008	UP
**GPX**	TRG	0.7813	4.721	2.049 - 8.303	1.654 - 10.252	0.012	UP
**TNF-α**	TRG	0.8075	91.592	46.152 - 153.749	37.888 - 166.887	0.002	UP

P(H1): Probability of alternative hypothesis that difference between untreated diabetic and control groups is due only to chance

TRG: Target; REF: Reference β-actin: Beta-actin, (Cu-Zn) SOD: Cu-Zn Superoxide Dismutase, GPX: Glutathione Peroxidase, TNF-α: Tumor Necrosis Factor Alpha

**Table 4:** Output data from Rest-RG software revealed the level of renal gene expression of Cu-Zn SOD, GPX, and TNF-α between control and HTD groups. The level of significant difference against the control group was set at *P-value<*0.05

**Gene**	**Type**	**Reaction efficiency**	**Expression**	**Std. Error**	**95% C.I.**	**P(H1)**	**Result**
**β-actin**	REF	0.775	1.000				
**Cu-Zn SOD**	TRG	0.7538	0.972	0.544 - 1.919	0.376 - 2.942	0.914	NSD
**GPX**	TRG	0.8	1.407	0.765 - 3.316	0.329 - 4.402	0.446	NSD
**TNF-α**	TRG	0.7813	0.019	0.010 - 0.038	0.006 - 0.054	0.028	DOWN

P(H1): Probability of alternative hypothesis that difference between histidine treated and control groups is due only to chance; TRG: Target; REF: Reference; NSD: No significant difference; β-actin: Beta-actin, (Cu-Zn) SOD: Cu-Zn Superoxide Dismutase, GPX: Glutathione Peroxidase, TNF-α: Tumor Necrosis Factor Alpha

## Conclusion

Our biochemical, molecular, and histological evaluations indicated that His could ameliorate diabetic complications, including hyperglycemia, dyslipidemia, and DN in type 2 diabetic rats. These ameliorative effects could be related to the improvement of insulin resistance, suppression of oxidative stress and inflammation through mitigation of LPO, restoration of anti-oxidant enzymes (activity & mRNA), and the decreased TNF-α mRNA level and MPO activity. The results of the present study revealed that due to its anti-oxidant and anti-inflammatory properties, His could ameliorate hyperglycemia, insulin resistance and secretion, dyslipidemia, oxidative stress, inflammation, and DN in the animal model of T2D. Hence, the use of His is recommended for diabetic patients in order to reduce diabetes complications.

## References

[1] Chen L, Magliano DJ, Zimmet PZ (2011). The worldwide epidemiology of type 2 diabetes mellitus--present and future perspectives. Nat Rev Endocrinol.

[2] Guariguata L, Whiting DR, Hambleton I, Beagley J, Linnenkamp U, Shaw JE (2014). Global estimates of diabetes prevalence for 2013 and projections for 2035. Diabetes Res Clin Pract.

[3] Hajivandi A, Amiri M (2013). World diabetes day 2013: diabetes mellitus and nephrology. J nephropharmacol.

[4] Brownlee M (2001). Biochemistry and molecular cell biology of diabetic complications. Nature.

[5] Amiri M (2018). Oxidative stress and free radicals in liver and kidney diseases; an updated short-review. J Nephropathol.

[6] Rodrigo R, Bosco C (2006). Oxidative stress and protective effects of polyphenols: comparative studies in human and rodent kidney A review. Comp Biochem Physiol C Toxicol Pharmacol.

[7] Shin AH, Oh CJ, Park JW (2006). Glycation-induced inactivation of anti-oxidant enzymes and modulation of cellular redox status in lens cells. Arch Pharm Res.

[8] Liu WH, Hei ZQ, Nie H, Tang FT, Huang HQ, Li XJ (2008). Berberine ameliorates renal injury in streptozotocin-induced diabetic rats by suppression of both oxidative stress and aldose reductase. Chin Med J (Engl).

[9] Tamadon MR, Zahmatkesh M, Beladi Mousavi SS (2015). Administration of anti-oxidants in chronic kidney disease. J Nephropharmacolo.

[10] Hasanvand A, Pirzadroozbahani N, Ahmadizar F, Kharazmkia A, Mir S, Baharvand P (2018). Evaluation of the anti-oxidant effects of zolpidem in the rat model of cisplatin-induced nephrotoxicity. Journal of Renal Injury Prevention.

[11] Bhattacharya S, Manna P, Gachhui R, Sil PC (2013). D-saccharic acid 1,4-lactone protects diabetic rat kidney by ameliorating hyperglycemia-mediated oxidative stress and renal inflammatory cytokines via NF-kappaB and PKC signaling. Toxicol Appl Pharmacol.

[12] Lee BT, Ahmed FA, Hamm LL, Teran FJ, Chen CS, Liu Y (2015). Association of C-reactive protein, tumor necrosis factor-alpha, and interleukin-6 with chronic kidney disease. BMC Nephrol.

[13] Roshan B, Stanton RC (2013). A story of microalbuminuria and diabetic nephropathy. J Nephropathol.

[14] Bahmani F, Bathaie SZ, Aldavood SJ, Ghahghaei A (2012). Glycine therapy inhibits the progression of cataract in streptozotocin-induced diabetic rats. Mol Vis.

[15] Jafarnejad A, Bathaie SZ, Nakhjavani M, Hassan MZ, Banasadegh S (2008). The improvement effect of L-Lys as a chemical chaperone on STZ-induced diabetic rats, protein structure and function. Diabetes Metab Res Rev.

[16] Feng RN, Niu YC, Sun XW, Li Q, Zhao C, Wang C (2013). Histidine supplementation improves insulin resistance through suppressed inflammation in obese women with the metabolic syndrome: a randomised controlled trial. Diabetologia.

[17] Jiang WD, Qu B, Feng L, Jiang J, Kuang SY, Wu P (2016). Histidine prevents Cu-induced oxidative stress and the associated decreases in mRNA from encoding tight junction proteins in the intestine of grass carp (Ctenopharyngodon idella). PLoS One.

[18] Abe H (2000). Role of histidine-related compounds as intracellular proton buffering constituents in vertebrate muscle. Biochemistry (Mosc).

[19] Lee YT, Hsu CC, Lin MH, Liu KS, Yin MC (2005). Histidine and carnosine delay diabetic deterioration in mice and protect human low density lipoprotein against oxidation and glycation. Eur J Pharmacol.

[20] Watanabe M, Suliman ME, Qureshi AR, Garcia-Lopez E, Barany P, Heimburger O (2008). Consequences of low plasma histidine in chronic kidney disease patients: associations with inflammation, oxidative stress, and mortality. Am J Clin Nutr.

[21] Masiello P, Broca C, Gross R, Roye M, Manteghetti M, Hillaire-Buys D (1998). Experimental NIDDM: development of a new model in adult rats administered streptozotocin and nicotinamide. Diabetes.

[22] Satheesh MA, Pari L (2008). Effect of pterostilbene on lipids and lipid profiles in streptozotocin-nicotinamide induced type 2 diabetes mellitus. J Appl Biomed (De Gruyter Open).

[23] Friedewald WT, Levy RI, Fredrickson DS (1972). Estimation of the concentration of low-density lipoprotein cholesterol in plasma, without use of the preparative ultracentrifuge. Clin Chem.

[24] Ikewuchi C (2009). Alteration of plasma lipid profiles and atherogenic indices by Stachytarpheta jamaicensis L(Vahl). Biokemistri.

[25] Ahmadvand H, Shahsavari G, Tavafi M, Bagheri S, Moradkhani MR, Kkorramabadi RM (2017). Protective effects of oleuropein against renal injury oxidative damage in alloxan-induced diabetic rats; a histological and biochemical study. J Nephropathol.

[26] Tavafi M, Ahmadvand H, Khalatbari A, Tamjidipoor A (2011). Rosmarinic acid ameliorates diabetic nephropathy in uninephrectomized diabetic rats. Iran J Basic Med Sci.

[27] Ahmadvand H, Tavafi M (2014). Amelioration of glomerulosclerosis by Satureja khozestanica essential oil in alloxan-induced diabetic rats. ZJRMS.

[28] Mahdavifard S, Bathaie SZ, Nakhjavani M, Heidarzadeh H (2014). l-cysteine is a potent inhibitor of protein glycation on both albumin and LDL, and prevents the diabetic complications in diabetic–atherosclerotic rat. Food Res Int.

[29] Doi M, Yamaoka I, Nakayama M, Sugahara K, Yoshizawa F (2007). Hypoglycemic effect of isoleucine involves increased muscle glucose uptake and whole body glucose oxidation and decreased hepatic gluconeogenesis. Am J Physiol Endocrinol Metab.

[30] Kimura K, Nakamura Y, Inaba Y, Matsumoto M, Kido Y, Asahara S (2013). Histidine augments the suppression of hepatic glucose production by central insulin action. Diabetes.

[31] Wijekoon EP, Skinner C, Brosnan ME, Brosnan JT (2004). Amino acid metabolism in the Zucker diabetic fatty rat: effects of insulin resistance and of type 2 diabetes. Can J Physiol Pharmacol.

[32] Rouhi H, Ganji F (2013). Effects of N-acetyl cysteine on serum lipoprotein (a) and proteinuria in type 2 diabetic patients. J Nephropathol.

[33] Mong MC, Chao CY, Yin MC (2011). Histidine and carnosine alleviated hepatic steatosis in mice consumed high saturated fat diet. Eur J Pharmacol.

[34] Borsheim E, Bui QU, Tissier S, Cree MG, Ronsen O, Morio B (2009). Amino acid supplementation decreases plasma and liver triacylglycerols in elderly. Nutrition.

[35] Jamor P, Ahmadvand H, Ashoory H, Babaeenezhad E (2019). Effect of alpha-lipoic acid on anti-oxidant gene expression and kidney injury in alloxan-induced diabetic rats. J Nephropathol.

[36] Yatzidis H (2004). Oral supplement of six selective amino acids arrest progression renal failure in uremic patients. Int Urol Nephrol.

[37] Sheela N, Jose MA, Sathyamurthy D, Kumar BN (2013). Effect of silymarin on streptozotocin-nicotinamide-induced type 2 diabetic nephropathy in rats. Iran J Kidney Dis.

[38] Faddah LM, Abdel Baky NA, Al-Rasheed NM, Al-Rasheed NM, Fatani AJ, Atteya M (2012). Role of quercetin and arginine in ameliorating nano zinc oxide-induced nephrotoxicity in rats. BMC Complement Altern Med.

[39] Sadar S, Kaspate D, Vyawahare N (2016). Protective effect of L-glutamine against diabetes-induced nephropathy in experimental animal: Role of KIM-1, NGAL, TGF-beta1, and collagen-1. Ren Fail.

[40] Arivazhagan P, Thilakavathy T, Panneerselvam C (2000). Antioxidant lipoate and tissue anti-oxidants in aged rats. J Nutr Biochem.

[41] Ahmadvand H, Tavafi M, Khosrowbeygi A (2012). Amelioration of altered anti-oxidant enzymes activity and glomerulosclerosis by coenzyme Q10 in alloxan-induced diabetic rats. J Diabetes Complications.

[42] Aboonabi A, Rahmat A, Othman F (2014). Anti-oxidant effect of pomegranate against streptozotocin-nicotinamide generated oxidative stress induced diabetic rats. Toxicol Rep.

[43] Yan SL, Wu ST, Yin MC, Chen HT, Chen HC (2009). Protective effects from carnosine and histidine on acetaminophen-induced liver injury. J Food Sci.

[44] Watanabe M, Nakashima H, Mochizuki S, Abe Y, Ishimura A, Ito K (2009). Amelioration of diabetic nephropathy in OLETF rats by prostaglandin I(2) analog, beraprost sodium. Am J Nephrol.

[45] Cheng H, Harris RC (2014). Renal endothelial dysfunction in diabetic nephropathy. Cardiovasc Hematol Disord Drug Targets.

[46] Gorudko I, Kostevich A, Sokolov A, Konstatinova E, Tsapaeva N, Mironova E (2012). Increased myelopepoxidase activity is a risk factor for ishemic heart disease in patients with diabetes mellitus. Biomeditsinskaia khimiia.

[47] Vita JA, Brennan ML, Gokce N, Mann SA, Goormastic M, Shishehbor MH (2004). Serum myeloperoxidase levels independently predict endothelial dysfunction in humans. Circulation.

[48] Madar Z, Kalet-Litman S, Stark AH (2005). Inducible nitric oxide synthase activity and expression in liver and hepatocytes of diabetic rats. Pharmacology.

[49] Zhang H, Tsao RJCOiFS (2016). Dietary polyphenols, oxidative stress and anti-oxidant and anti-inflammatory effects.

[50] Yagmurca M, Erdogan H, Iraz M, Songur A, Ucar M, Fadillioglu E (2004). Caffeic acid phenethyl ester as a protective agent against doxorubicin nephrotoxicity in rats. Clin Chim Acta.

[51] Todorova VK, Kaufmann Y, Hennings L, Klimberg VS (2010). Oral glutamine protects against acute doxorubicin-induced cardiotoxicity of tumor-bearing rats. J Nutr.

[52] Navarro JF, Milena FJ, Mora C, Leon C, Garcia J (2006). Renal pro-inflammatory cytokine gene expression in diabetic nephropathy: effect of angiotensin-converting enzyme inhibition and pentoxifylline administration. Am J Nephrol.

[53] Andou A, Hisamatsu T, Okamoto S, Chinen H, Kamada N, Kobayashi T (2009). Dietary histidine ameliorates murine colitis by inhibition of proinflammatory cytokine production from macrophages. Gastroenterology.

[54] Sadi G, Eryilmaz N, Tutuncuoglu E, Cingir S, Guray T (2012). Changes in expression profiles of anti-oxidant enzymes in diabetic rat kidneys. Diabetes Metab Res Rev.

[55] Matsunami T, Sato Y, Sato T, Ariga S, Shimomura T, Yukawa M (2010). Oxidative stress and gene expression of anti-oxidant enzymes in the streptozotocin-induced diabetic rats under hyperbaric oxygen exposure. Int J Clin Exp Pathol.

[56] Cao C, Leng Y, Huang W, Liu X, Kufe D (2003). Glutathione peroxidase 1 is regulated by the c-Abl and Arg tyrosine kinases. J Biol Chem.

[57] Reddi AS, Bollineni JS (1997). Renal cortical expression of mRNAs for anti-oxidant enzymes in normal and diabetic rats. Biochem Biophys Res Commun.

[58] Limaye PV, Raghuram N, Sivakami S (2003). Oxidative stress and gene expression of anti-oxidant enzymes in the renal cortex of streptozotocin-induced diabetic rats. Mol Cell Biochem.

